# Modeling the Effects of Duration and Size of the Control Zones on the Consequences of a Hypothetical African Swine Fever Epidemic in Denmark

**DOI:** 10.3389/fvets.2018.00049

**Published:** 2018-03-19

**Authors:** Tariq Halasa, Anette Bøtner, Sten Mortensen, Hanne Christensen, Sisse Birk Wulff, Anette Boklund

**Affiliations:** ^1^National Veterinary Institute, Technical University of Denmark, Lyngby, Denmark; ^2^Danish Veterinary and Food Administration, Ministry of Environment and Food, Glostrup, Denmark

**Keywords:** African swine fever, model, simulation, control, surveillance

## Abstract

African swine fever (ASF) is a notifiable infectious disease. The disease is endemic in certain regions in Eastern Europe constituting a risk of ASF spread toward Western Europe. Therefore, as part of contingency planning, it is important to continuously explore strategies that can effectively control an epidemic of ASF. A previously published and well documented simulation model for ASF virus spread between herds was used to examine the epidemiologic and economic impacts of the duration and size of the control zones around affected herds. In the current study, scenarios were run, where the duration of the protection and surveillance zones were reduced from 50 and 45 days to 35 and 25 days or to 35 and 25 days, respectively. These scenarios were run with or without enlargement of the surveillance zone around detected herds from 10 to 15 km. The scenarios were also run with only clinical or clinical and serological surveillance of herds within the zones. Sensitivity analysis was conducted on influential input parameters in the model. The model predicts that reducing the duration of the protection and surveillance zones has no impact on the epidemiological consequences of the epidemics, while it may result in a substantial reduction in the total economic losses. In addition, the model predicts that increasing the size of the surveillance zone from 10 to 15 km may reduce both the epidemic duration and the total economic losses, in case of large epidemics. The ranking of the control strategies by the total costs of the epidemics was not influenced by changes of input parameters in the sensitivity analyses.

## Introduction

African swine fever (ASF) is caused by ASF virus (ASFV), a DNA virus within the family Asfarviridae, genus *Asfivirus* (1). Since its introduction into Georgia in 2007, the virus spread to several countries including the Russian Federation, and several Eastern European Union (EU) countries (2), including recently the Czech republic (3). This situation poses a serious risk of spread of ASFV to Western European countries (4). Thus, it is important to continuously explore cost-effective strategies to control outbreaks of ASF in the industrialized swine populations.

European Union initially established a set of strategies, which should be followed in the case of an outbreak of ASF in the domestic swine populations, including the culling of all susceptible animals on detected premises followed by cleaning and disinfection (5). These strategies were amended later by adding extra control actions such as disinfection of vehicles, suspension of markets, stricter biosecurity, and other actions (6). One of the important actions is the establishment of the protection and surveillance zones around the affected herds directly after detection, in which all herds with susceptible animal species are surveyed (5). The duration of these control zones should be at least 45 days for the protection zone and 40 days for the surveillance zone (6). This long duration has negative impact on the intra-community trade (within the EU) and export of swine and swine products from the zones and from the whole country. However, the EU allows shorter duration of the zones, if intensive surveillance within these control zones is carried out (5). As the presence of zones has a negative impact on the export of swine and swine products from the affected countries, it might reduce the economic costs of epidemics if the duration of the zones is shortened. It is important to ensure that reducing the duration of the zones does not jeopardize the epidemiological consequences of the epidemics leading to extra economic negative effects. In the European Commission African swine fever diagnostic manual (5), intensive surveillance is defined as sampling for the detection of 5% seroprevalence with 95% confidence in each subunit of all holdings. An alternative method for intensive surveillance of all holdings in control zones was proposed by testing of up to five dead pigs per herd per week for detection of ASF genome within the control zones (7).

In addition, increasing the size of the surveillance zone might improve the control of the disease, as more herds around the infected herds are surveyed, and hence the infected ones have higher chance to be detected. To our knowledge, this has never been explored before.

The objective of this study was to investigate the epidemiological and economic impact of shorter duration of control zones with or without increasing the size of the surveillance zone during a hypothetical epidemic of ASF in an industrialized swine population using the Danish swine population as an example.

## Materials and Methods

### Herd and Movement Data

We used geographical data (UTM coordinates), the number of animals, and specification of herd types for the 8,262 swine herds registered in the Danish Central Husbandry Register in 2014. For each herd, the daily frequency of outgoing animal movements (in batches) was used as the mean (λ) in a Poisson distribution, describing the number of daily out-going movements (see Table S1 in Supplementary Material). Similarly, the probability of moving animals to an abattoir was calculated for each herd. Separate distributions for movement distances were used to model the movements of animals from nucleus herds and from other herd types (8).

### The Simulation Model

The DTU-DADS-ASF model version 0.15.1, which is a buildup from the original DTU-DADS model (9, 10) was used for this study (8, 11). The model is available at https://github.com/THalasa/DTU-DADS-ASF. The model runs in the statistical computing language R (version 3.1.3) (12). The model included a minor update to version 0.15.2. In the earlier version of the model, the expected number of dead animals due to other reasons than ASF was fixed over time. The update allows variable number of dead animals over time, which is more realistic. It also estimated an approximation of the number of submitted samples from surveillance of dead animals after the end of the epidemics.

#### Modeling ASF Spread

ASF virus spread was modeled in two processes: (1) spread within a herd and (2) spread between herds *via* several mechanisms.

##### Modeling ASFV Spread Within a Herd

The infection model for the individual animals is a state transition model with the following states: susceptible-latent-subclinical-clinical-removed (SLSCR model) (11). Infected pigs become latent in which virus is not shed. Thereafter, they become subclinical, where they do not show clinical symptoms, but have the potential to shed the virus. Once they enter the clinical stage, they show clinical symptoms and are fully infectious. 95% of the clinical cases die, while the rest survive and become immune. The duration of each stage is presented in Figure S1 in Supplementary Material. Other parameters related to the disease including the transmission rates are presented in Halasa et al. (8) and Table S2 in Supplementary Material. Infected herds follow the same dynamics, but with the possibility to become susceptible again should the disease fade out. Infected herds may, as explained beneath, be detected, and hence culled, or recovered.

##### Modeling ASFV Spread Between Herds

The virus may spread between herds *via* animal movements between herds, *via* abattoir trucks, *via* indirect medium-risk contacts (e.g., veterinarians or artificial inseminators with direct contact to animals) or low-risk contacts (e.g., feed trucks and visitors with no direct contacts to animals), or *via* local spread (8). For movements of animals (high-risk contacts) to other herds or to abattoirs, the frequency of contact (λ) for the individual herds was used in a Poisson distribution. For indirect medium (contacts that include direct contact to animals, such as contacts by veterinarians) and low (contacts that do not necessarily include direct contact to animals such as contacts by feeding trucks) risk contacts, the frequency of contact (λ) was modeled for each herd type. The probability of transmitting ASFV from the infectious herd to the receiving herd was dependent on the prevalence of the disease within the infectious herd and the number of animals moved in the batch (8). Local spread was modeled for distances up to 2 km around infectious herds and was assumed to consist of a mixture of unregistered animal movements, shared equipment and tools, and spread *via* rodents and insects. Detailed information including the equations and steps for modeling each of these mechanisms can be found in Halasa et al. (8).

The risk of ASFV spread and/or maintenance through wild boar was not modeled, as the number of wild boar in Denmark is limited due to intensive farming in the country, leaving few suitable habitats for wild boar (13, 14). Also, there is a Danish legal requirement to eliminate stray wild boar (15).

#### Modeling ASFV Detection

In the model, the ASFV infection can be detected by three different mechanisms: passive surveillance before first detection; passive surveillance after first detection; and active surveillance.

For passive surveillance, before first detection, diagnosis of ASFV infection was modeled to occur when (1) the cumulative proportion of sick or dead animals (referred to as SIED throughout the paper) reached 2.55% (8); (2) in the period from the appearance of ASF clinical signs until the current time step, the proportion of SIED animals relative to the expected cumulative mortality level within the herd had increased by 2 (8); and (3) the number of SIED animals within the herd reached five animals (8). For passive surveillance, after first detection, the first two conditions were assumed to be the same as before first detection, while the minimum number of SIED animals was set to 1, to represent a higher awareness of the disease in the country (7, 8).

For active surveillance, detection occurred as a result of surveillance visits to herds by official veterinarians, either due to tracing or because the herd was located in a control zone (7, 8). The active surveillance includes either clinical surveillance alone (clinical signs and mortality), or clinical surveillance combined with serological and/or PCR testing, depending on the control strategy modeled. In case of clinical surveillance only, suspicion was assumed to occur, if points 2 and 3 (in passive surveillance before first detection) were reached. Suspicions were then followed up by serological and/or PCR testing for confirmation of ASFV (7, 8).

We used these conditions based on the cumulative SIED to account mechanistically for the variation in time from infection to detection between herds, which is expected to occur due to the variation in spread of virus with the infected herds.

#### Modeling Control Strategies

In our earlier work (7), we have predicted the application of the basic EU and Danish control strategies (6, 16–18) combined with surveillance of dead animals in herds within the control zones (protection and surveillance zones) to be the most cost-effective scenario of the compared control strategies. Thus, the basic control strategies in this study included: (1) culling, cleaning, and disinfection of affected herds; (2) a 3-day national standstill on animal movements; (3) establishment of protection zone of minimum 3 km and a surveillance zone of minimum 10 km surrounding the affected herds; (4) tracing of movements and contacts; and (5) surveillance and testing of up to five dead pigs per herd per week within the surveillance and protection zones.

Within both control zones, movements must be restricted and herds within the zones must be surveyed for ASF. Herds within the protection zone were simulated to be visited twice; a first visit quickly after establishment of the zone including clinical surveillance only and a second visit before lifting the zone including serological testing. Herds in the surveillance zone were assumed to be visited only once before lifting the zone, including clinical surveillance only. The duration of the protection and surveillance zones was simulated to be 50 and 45 days, respectively.

Surveillance and testing of dead animals includes PCR and serological testing of one to five dead animals per herd per week from all herds in these control zones during the full duration of the zones.

In this study, besides the basic control scenario “Basic” that is described above, we simulated seven alternative control scenarios: (1) The same as “Basic,” but with increased size of the surveillance zone from 10 to 15 km (“Basic + LZ”); (2) The same as “Basic,” but with reduced duration of the protection and surveillance zones from 50 and 45 days to 35 and 35 days, respectively (“Red.ZD1”); (3) The same as “Red.ZD1,” but with increased size of the surveillance zone from 10 to 15 km (“Red.ZD1 + LZ”); (4) The same as “Basic,” but with reduced duration of the protection and surveillance zones from 50 and 45 days to 35 and 25 days, respectively (“Red.ZD2”); (5) The same as “Red.ZD2,” but with increased size of the surveillance zone from 10 to 15 km (“Red.ZD2 + LZ”); (6) The same as “Red.ZD2,” but adding serological testing of herds in the surveillance zone before lifting the zone, instead of clinical surveillance only (“Red.SZ2 + Ser”); and (7) The same as “Red.SZ2 + Ser,” but with increased size of the surveillance zone from 10 to 15 km (“Red.SZ2 + Ser + LZ”).

#### Model Outcomes and Run

The model provides per iteration epidemiological and economic outcomes. The epidemiological outcomes include epidemic duration, number of infected, detected, surveyed, and culled herds. In addition, the model provided outcomes on animal level such as the number of culled and surveyed animals. The economic outcomes include the direct costs, export, and total losses. The direct costs included surveillance, depopulation, cleaning and disinfection, and compensation. In addition, costs of empty stables, costs of welfare slaughter, costs of a 3 days national standstill and costs of samples submission and analysis for surveillance of dead animals were included. The *export losses* included export bans on livestock and livestock products to EU and non-EU countries. The losses were calculated for live pigs as well as pig products and were divided into losses from export bans on: live swine to EU countries, swine products to EU countries, and live swine and swine products to non-EU countries. Detailed information on the economic analysis and input parameters are presented in Halasa et al. (8).

Each scenario was run 2,000 iterations. Following visual inspection of model convergence, this number of iterations was deemed sufficient to obtain stable outcomes (8).

#### Sensitivity Analysis

Sensitivity and robustness analyzes on model input parameters were conducted earlier (7, 8, 11) showing a high robustness of the model predictions. The analyses identified the transmission rate of the virus and the proportion of SIED animals to be potentially influential parameters. Thus in this study, we examine, whether the ranking by the total costs of the epidemics of the simulated scenarios will change following changes in the values of these potentially influential parameters by 25% increase or reduction. The original transmission rates of the virus were drawn from random PERT distributions with minimum 0.14, mode 0.38 and maximum 0.8 for production and nucleus herds, and minimum 0.36, mode 0.60 and maximum 0.93 for the other herd types (8).

## Results

**Figure 1:**
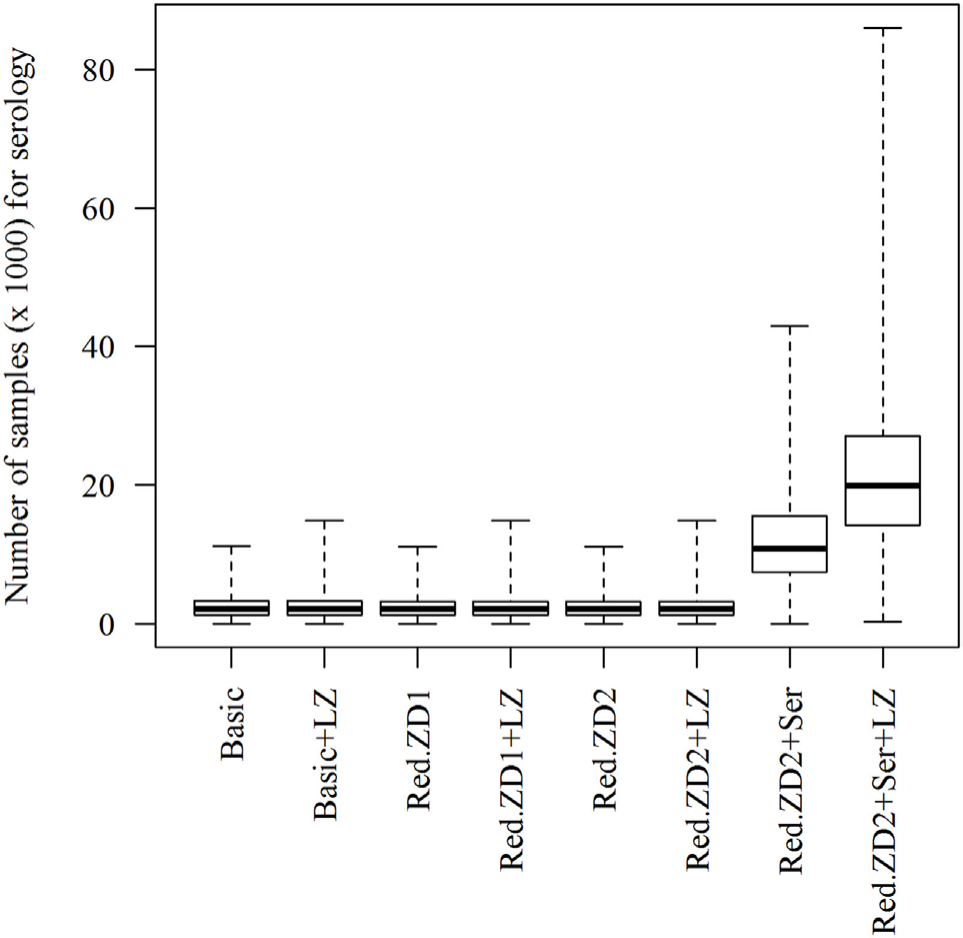
Predicted number of samples (animal-samples) for serological testing when different control scenarios against African swine fever-epidemics are simulated. Basic: (1) culling, cleaning, and disinfection of affected herds; (2) 3-day national standstill on animal movements; (3) 3-km protection zone—duration 50 days + 10-km surveillance zone duration 45 days (movements and contact restrictions in zones); and (4) tracing of movements and contacts. Basic + LZ: basic control + surveillance zone 15 km (default: 10 km). Red.ZD1: Basic + duration of protection and surveillance zones 35 days (default: 50 and 45 days). Red.ZD1 + LZ: Red.ZD1 + surveillance zone 15 km (default: 10 km). Red.ZD2: Basic + duration of protection and surveillance zones 35 and 25 days, respectively (default: 50 and 45 days). Red.ZD2 + LZ: Red.ZD2 + surveillance zone 15 km (default 10 km). Red.ZD2 + Ser: Red.ZD2 + serological testing in surveillance zone before lifting the zone (default: clinical surveillance). Red.ZD2 + Ser + LZ: Red.ZD2 + Ser + surveillance zone 15 km (default: 10 km).

When the “Basic” scenario was simulated, duration of an epidemic of 9 days (median value) is predicted, with variation from 1 to 39 days (5th and 95th percentiles) (Table 1). Furthermore, infection of three herds (median value), with variation from one to eight herds (5th and 95th percentiles) is predicted (Table 1). Most often, all herds were predicted to be detected and culled (Table 1). One herd (5th–95th percentiles: 0–3) is predicted to be detected *via* surveillance of dead animals from holdings in the control zones. In total, an economic loss of €296 million (€258–397 million) is predicted. Reducing the duration of the protection and surveillance zones from 50 and 45 days to 35 days for both (Red.ZD1) has no impact on the epidemics compared to the basic scenario. It would though result in fewer numbers of events for surveillance of dead animals and in a substantial reduction of the total losses due to reduction in export losses (Table 1). This was also the case, when the duration of the protection and surveillance zones was reduced from 50 and 45 days to 35 and 25 days, respectively (Red.ZD2). Adding serological surveillance for herds in the surveillance zones before lifting the zone to this scenario (Red.SZ2 + Ser) was not predicted to further reduce the size, duration, or economic consequences of the epidemics, while a substantial increase in the number of samples for serology testing was predicted (Figure 1).

**Table 1:** Median and 5th and 95th percentiles of predicted epidemiological and economic consequences of controlling a hypothetical epidemic of African swine fever in Denmark, using three different control scenarios.

	Basic	Basic + LZ	Red.ZD1	Red.ZD1 + LZ	Red.ZD2	Red.ZD2 + LZ	Red.ZD2 + Ser	Red.ZD2 + Ser + LZ
Epidemic duration (days)	9 (1–39)	9 (1–36)	9 (1–39)	9 (1–36)	9 (1–39)	9 (1–35)	9 (1–39)	9 (1–35)
Infected herds	3 (1–8)	3 (1–8)	3 (1–8)	3 (1–8)	3 (1–8)	3 (1–8)	3 (1–8)	3 (1–8)
Detected herds	3 (1–7)	3 (1–7)	3 (1–7)	3 (1–7)	3 (1–7)	3 (1–7)	3 (1–7)	3 (1–7)
Culled herds	3 (1–7)	3 (1–7)	3 (1–7)	3 (1–7)	3 (1–7)	3 (1–7)	3 (1–7)	3 (1–7)
Herds detected from active surveillance	0 (0–2)	0 (0–2)	0 (0–2)	0 (0–2)	0 (0–2)	0 (0–2)	0 (0–2)	0 (0–2)
Herds detected from surveillance of dead animals	1 (0–3)	1 (0–3)	1 (0–3)	1 (0–3)	1 (0–3)	1 (0–3)	1 (0–3)	1 (0–3)
Clinical surveillance only (herd level)	111 (38–283)	206 (81–494)	111 (38–289)	205 (81–509)	111 (38–293)	206 (81–538)	18 (5–156)	17 (5–259)
Serology surveillance (herd level)	19 (5–55)	19 (5–54)	19 (5–53)	19 (5–51)	19 (5–54)	19 (5–51)	103 (37–242)	195 (81–410)
PCR surveillance (herd level)	3 (0–8)	3 (0–8)	3 (0–8)	3 (0–8)	3 (0–8)	3 (0–8)	3 (0–8)	3 (0–8)
Surveillance of dead animals (herd level)	455 (134–1,248)	846 (272–2,192)	333 (98–950)	625 (203–1,668)	331 (98–926)	622 (203–1,611)	331 (98–926)	622 (203–1,611)
Direct costs (€ million)	9 (7–15)	11 (8–19)	8 (6–12)	9 (7–15)	8 (6–12)	9 (7–15)	8 (6–13)	10 (7–15)
Export losses (€ million)	287 (250–383)	290 (254–386)	254 (218–348)	256 (220–348)	254 (218–348)	256 (220–348)	254 (218–348)	256 (220–348)
Total losses (€ million)	296 (258–397)	301 (264–401)	262 (225–360)	265 (228–361)	262 (225–360)	265 (228–361)	262 (225–360)	265 (228–361)

An increase in the size of the surveillance zone from 10 to 15 km is not predicted to improve the control of the epidemics and would not pay off the extra costs, under the median situation (Table 1). Nevertheless, in case of larger epidemics (the 95th percentile), an increased size of this zone is predicted to result in slightly shorter epidemic duration, and total costs close to similar scenarios with the original zone-size. This indicates that increasing the size of the surveillance zone may be beneficial to improve the control of large ASF-epidemics.

**Figure 2:**
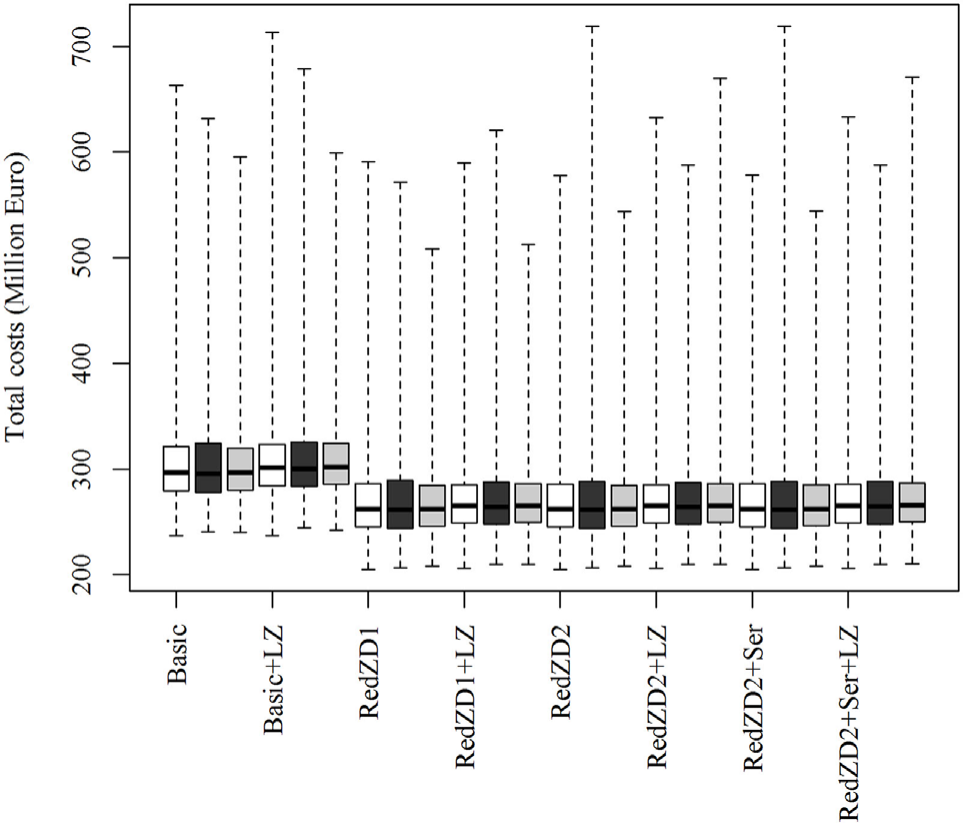
Box plots of the predicted total costs of African swine fever-epidemics using different control scenarios with changed values of the proportion of sick and dead animals (SIED) needed for detection of infected herds to occur. The empty, black and gray boxes represent the control scenario with the default, reduced and increased SIED value, respectively. Basic: (1) culling, cleaning, and disinfection of affected herds; (2) 3-day national standstill on animal movements; (3) 3-km protection zone—duration 50 days + 10-km surveillance zone duration 45 days (movements and contact restrictions in zones); and (4) tracing of movements and contacts. Basic + LZ: basic control + surveillance zone 15 km (default: 10 km). Red.ZD1: Basic + duration of protection and surveillance zones 35 days (default: 50 and 45 days). Red.ZD1 + LZ: Red.ZD1 + surveillance zone 15 km (default: 10 km). Red.ZD2: Basic + duration of protection and surveillance zones 35 and 25 days, respectively (default: 50 and 45 days). Red.ZD2 + LZ: Red.ZD2 + surveillance zone 15 km (default 10 km). Red.ZD2 + Ser: Red.ZD2 + serological testing in surveillance zone before lifting the zone (default: clinical surveillance). Red.ZD2 + Ser + LZ: Red.ZD2 + Ser + surveillance zone 15 km (default:10 km).

**Figure 3:**
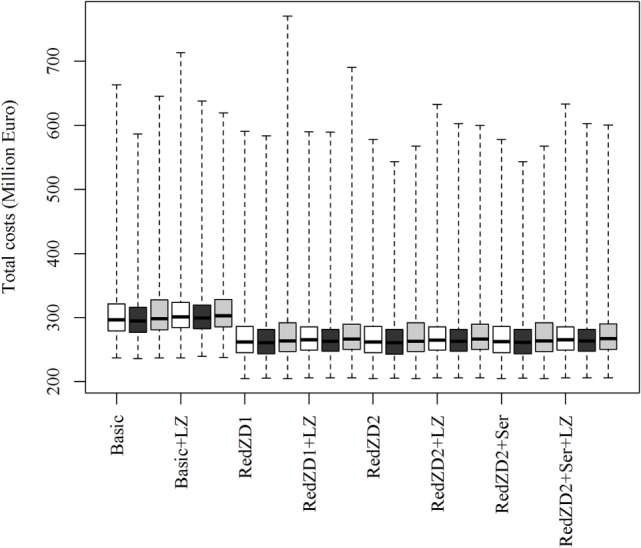
Box plots of the predicted total costs of African swine fever-epidemics using different control scenarios with changed values for the transmission rate of the virus. The empty, black and gray boxes represent the control scenario with the default, reduced and increased transmission rate value, respectively. Basic: (1) culling, cleaning, and disinfection of affected herds; (2) 3-day national standstill on animal movements; (3) 3-km protection zone—duration 50 days + 10-km surveillance zone duration 45 days (movements and contact restrictions in zones); and (4) tracing of movements and contacts. Basic + LZ: basic control + surveillance zone 15 km (default: 10 km). Red.ZD1: Basic + duration of protection and surveillance zones 35 days (default: 50 and 45 days). Red.ZD1 + LZ: Red.ZD1 + surveillance zone 15 km (default: 10 km). Red.ZD2: Basic + duration of protection and surveillance zones 35 and 25 days, respectively (default: 50 and 45 days). Red.ZD2 + LZ: Red.ZD2 + surveillance zone 15 km (default 10 km). Red.ZD2 + Ser: Red.ZD2 + serological testing in surveillance zone before lifting the zone (default: clinical surveillance). Red.ZD2 + Ser + LZ: Red.ZD2 + Ser + surveillance zone 15 km (default:10 km).

The ranking of the control strategies, compared by the total costs of the epidemics, is not predicted to be affected by neither changes in the proportion of dead animals in an infected herd needed for detection to occur, nor by changes in the transmission rate of the virus (Figure 2). Nevertheless, these changes may result in more extreme epidemics, where the losses become very large (Figures 2 and 3).

## Discussion

Since the introduction of ASFV to Georgia in 2007, the virus spread to the Russian Federation and several European countries (2). It also took the attention of the national veterinary authorities of Western European countries, especially those with large swine industries, in order to prevent its introduction into the countries. Increased emphasis was put on checking and updating the national contingency plans for the control of ASFV spread in case introduction should occur.

Clear regulations have been set by the EU for the control of ASF-outbreaks in pig holdings (5), and subsequently amended several times based on the recent experiences of the virus spread in Europe as discussed earlier (6). The regulations demand that the death of swine from herds within the protection and surveillance zones is reported and that herds in the protection and surveillance zones are surveyed for at least 45 and 40 days, respectively, before lifting the zones (5). For logistical reasons, we added extra days to each of the zones in the model, to make sure that all herds are visited, before the zones are lifted. Nevertheless, the regulations allow reduction of the duration of the protection and surveillance zones to 30 and 20 days, respectively, when intensive surveillance is implemented (5). In our previous work, we have shown that including intensive surveillance and diagnostic testing of dead animals from swine herds within the control zones improves the control of the epidemics substantially, and that testing of live animals would have only marginal impact on the control of the virus spread (7). This motivated the proposal of a reduced duration of the protection and surveillance zones from 50 and 45 days to 35 days for both (“Red.ZD1”) or to 35 and 25 days (“Red.ZD2”), respectively, which includes intensive surveillance of dead animals. These scenarios clearly show that the reduction of the duration of the control zones would not jeopardize the control actions and lead to extra spread of the virus (Table 1). Instead, they would lead to the same epidemiological consequences, but to substantially lower total economic losses compared to the basic scenario (Table 1). Because the vast majority of the detected herds from surveillance of dead animals are predicted to be detected in the first round of testing of the dead animals, repeated testing of dead animals does not seem to add extra value. This might not be the case in situations with fast spreading epidemics of larger size. The reduction in total losses is driven by the reduction in export losses (Table 1). Reducing the duration of the control zones allows faster recovery of export of swine and swine products leading to lower export losses. Adding serological surveillance of live animals from herds in the surveillance zone (“Red.ZD2 + Ser”) were predicted to affect neither the epidemiological nor the economic consequences of the epidemics (Table 1) and, therefore, has no added value in documenting freedom from ASF in control zones. This is consistent with our previous finding regarding serological surveillance of live animals (7). Nevertheless, this would result in a large number of samples for serological testing (Figure 1). This clearly warrants the investigation of resource capacities during an outbreak situation, including laboratory capacities as carried out earlier for FMD (9).

Earlier work has shown that increasing the size of the surveillance zone can improve the control of foot-and-mouth disease, as infected herds would be detected earlier and hence extra virus spread is prevented (10). This motivated testing the same scenarios in this study, but with increasing the size of the surveillance zone from 10 to 15 km. In general, because the epidemics are predicted to be small, the gain of increasing the size of the zone would not pay off the extra costs (Table 1). However, when the epidemics are large (as in case of the 95th percentiles), the gain of increasing the size of the zone, by having shorter epidemic duration may pay off the losses from extra surveyed herds (direct costs). Following from that, the increase of zone-size could be seen as an insurance against large epidemics.

Previous work have already pointed out the most sensitive parameters of the model and showed that model predictions were robust (8), and the conclusions regarding the optimal control scenarios would not change by changing the input parameter values (7). This was also the case in the current study confirming the robustness of the model predictions.

The current model can be applied for outbreaks in pig populations where wild boar are absent as in the case of Denmark or where wild boar are not involved in ASF-epidemics as in the recent outbreak in Romania (19). However, given the importance of wild boar in the spread of ASFV in Europe (6), there is a need to further develop simulation models to also include the involvement of ASF-outbreaks in wild boar.

## Conclusion

Reduction of the duration of the protection and surveillance zones, implemented during ASF-epidemics, was predicted to result in a substantial reduction in the total losses, while the epidemics were not affected. In addition, increasing the size of the surveillance zone from 10 to 15 km can be an insurance against large ASF-epidemics. This was based on simulation of ASF spread in an industrialized swine population without a wild boar population.

## Author Contributions

TH conducted the study and wrote the manuscript. All authors designed the study and approved the manuscript.

## Conflict of Interest Statement

The authors declare that the research was conducted in the absence of any commercial or financial relationships that could be construed as a potential conflict of interest.
