# Supplementary material for: Modeling the Effects of Duration and Size of the Control Zones on the Consequences of a Hypothetical African Swine Fever Epidemic in Denmark
Source: Front Vet Sci. 2018 Mar 19;5:49. doi: 10.3389/fvets.2018.00049 (PMC5868302; doi:10.3389/fvets.2018.00049)
Supplement: Supplementary file 1 [file data_sheet_1.DOCX]

Supplementary Material

**Modeling the effects of duration and size of the control zones on the consequences of a hypothetical African swine fever epidemic in Denmark**

**Tariq Halasa*, Anette Bøtner, Sten Mortensen, Hanne Christensen, Sisse Birk Wulff, Anette Boklund**

*** Correspondence:** Corresponding Author: tahbh@vet.dtu.dk

# Supplementary Figure and Tables

## Supplementary Figure


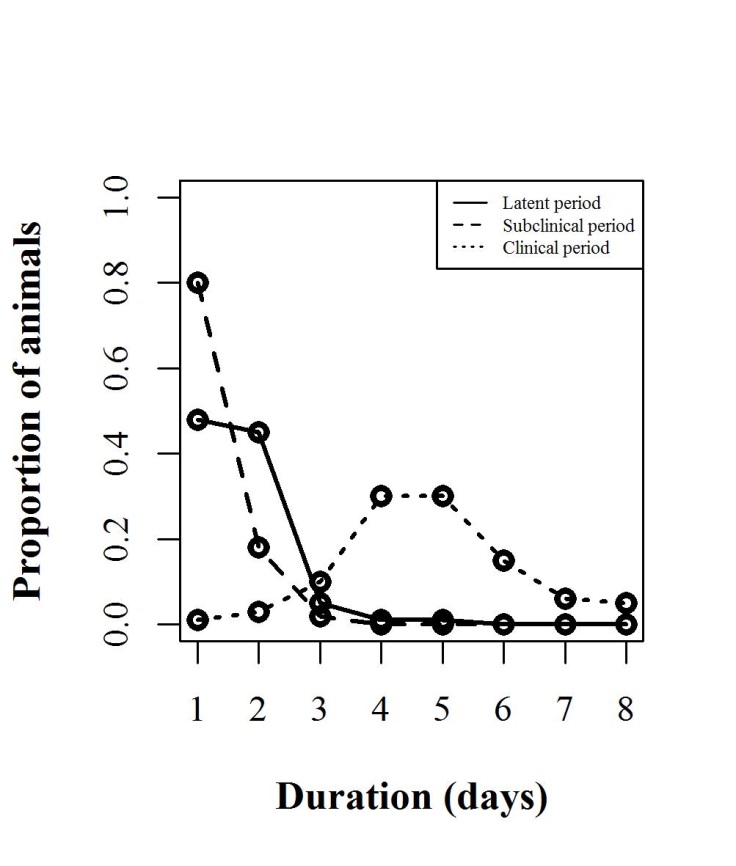


**Supplementary Figure S1**. Disease stages used for simulating African swine fever. Proportion of animals within a pig unit (y-axis) that will spend the defined number of days (x-axis) in each infection stage (latent, subclinical and clinical).

**Table S1**. Descriptive data of the 8262 swine herds classified on herd type.

| Herd types^1^ | Number of herds | Herd size,  median (5-95%) | Frequency of outgoing movements per day to other herds,  median (5-95%) | Frequency of outgoing movements per day to abattoir,  median (5-95%) |
| --- | --- | --- | --- | --- |
| Nucleus^2^ | 254 | 2200 (520-5991) | 0.1328 (0-0.4074) | 0.1233 (0.0027-0.3663) |
| Boar station^3^ | 18 | 200 (29-395) | 0 (0-0.1155) | 0.0329 (0-0.0666) |
| Quarantine^4^ | 53 | 80 (2-444) | 0.0082 (0-0.0778) | 0 (0-0) |
| Backyard^5^ | 1048 | 2 (2-5) | 0 (0-0) | 0 (0-0.0082) |
| SPF^6^ hobby^7^ | 5 | 73 (10-91) | 0.0082 (0-0.2880) | 0 (0-0.0307) |
| SPF conventional | 473 | 16 (6-104) | 0 (0-0.0027) | 0.0055 (0-0.0493) |

All herd types, except backyard and hobby, were based on the types stated in CHR

2 Defined as top breeding and multiplying herds, which deliver breeding stock for production herd, i.e. sows and boar for sow herds.

3 Boar stations produce semen

4 Herds where pigs are quarantined before entering other herds. Most often these are used for boars before they enter the boar stations.

5 ≤5 animals. Still, these animals are kept on farms, as Danish legislation states that all pigs must be fenced with double fencing or in stables.

6 Specific Pathogen Free herds. These herds are tested yearly or monthly for a number of diseases and are under strict regulation regarding biosecurity, for example with demands for changing facilities befor entering the farm and for pick-up of animals etc.

7 <20 sows, <100weaners and <100 finishers OR registered as HOBBY in CHR

**Table S2**. Parameters, values and assumptions used to parameterize the simulation model. The table is obtained from Halasa et al. (2016)

| Parameter description | | Value | | Reference | |
| --- | --- | --- | --- | --- | --- |
| Daily average number of animal shipments to other herds | | Poisson (λ_Herd-specific_) | | Movement data from Danish CHR and movement database for swine | |
| Daily average number of animal shipments to abattoir | | Poisson (λ_Herd-specific_) | | Movement data from Danish CHR and movement database for swine | |
| Daily average medium risk contacts (vets and AI) | | Lookup table: indoor: Poisson (λ = 0.036), outdoor: Poisson (λ = 0.0027) | | Boklund et al. (2013) | |
| Daily average low risk contacts (nonprofessional visitors and rendering and feed trucks) | | Lookup table: indoor: Poisson (λ = 0.22), outdoor: Poisson (λ = 0.1837), | | Boklund et al. (2013) | |
| Probability of infection via medium risk contact from an infectious herd | | Lookup table: indoor: PERT (min=0.01, mode=0.03,max=0.1), outdoor: PERT (min=0.01, mode=0.06, max=0.1) | | Adjusted according to Nigsch et al. (2013) based on Boklund et al. (2009) | |
| Probability of infection via low risk contact from an infectious herd | | Lookup table: indoor swine: PERT (min=0.005, mode=0.011,max=0.05), outdoor swine: PERT (min=0.005, mode=0.0175, max=0.05) | | Adjusted according to Nigsch et al. (2013) based on Boklund et al. (2009) | |
| Probability to become infected through local spread at a specific day given distance from an infectious premise | | Lookup table: from 0 to 0.1 km: 0.1, from 0.1 to 0.5 km: 0.006, from 0.5 to 1 km: 0.002, from 1 to 2km: 0.000015 | | Adjusted according to Nigsch et al. (2013) based on Boklund et al. (2009) | |
| Probability of disease transmission to Specific Pathogen free (SPF) herds | | 50% lower than other herds | | Boklund et al. (2009) | |
| Herd size categories | | Backyard: 2-5, Small herds: 6-300, Medium size herds**:** 301-1200, Large herds: 1201-2250, Very large herds: >2250 | | The Central Husbandry Register (CHR) data provided by the Veterinary, Authorities | |
| Number of moved animals per batch from a breeding herd for the different herd size categories | | Backyard**:** PERT (min=1,mode=1,max=2),  Small: PERT (min=1,mode=61,max=196),  Medium: PERT (min=1,mode=85,max=415),  Large: PERT(min=1,mode=110,max=433),  Very large: PERT (min=1,mode=81,max=371) | | Movement data from Danish CHR and movement database for swine | |
| Number of moved animals per batch from other herd types than breeding herds for the different herd size categories | | Backyard**:** PERT (min=1,mode=1,max=2),  Small: PERT (min=1,mode=25,max=290),  Medium: PERT (min=1,mode=304,max=800),  Large: PERT(min=1,mode=300,max=910),  Very large: PERT (min=1,mode=290,max=800) | | Movement data from Danish CHR and Movement database for swine | |
| Number of contact herds on the route of the abattoir truck before disease detection | | Exp (rate=1) | | Movement data for swine | |
| Number of contact herds on the route of the abattoir truck after disease detection | | Exp (rate=2) | | Assumed value to address 50% reduction of contacts following disease detection | |
| Capacity for depopulation per day | | 4800 animals | | Boklund et al. (2013) | |
| Daily capacity for surveillance | | First week = 50 herds  Second week = 100 herds  After second week = 200 herds | | The Danish Veterinary and Food Administration | |
| Probability of imposing a successful restrictions on animal movements within the protection and surveillance zones | | PERT (min=0.95, mode=0.98,max=1) | | Boklund et al. (2013) | |
| Probability of imposing a successful restrictions on medium risk contacts within the protection and surveillance zones | | PERT (min=0.7, mode=0.8,max=0.95) | | Boklund et al. (2013) | |
| Probability of imposing a successful restrictions on low risk contacts within the protection and surveillance zones | | PERT (min=0.2, mode=0.3,max=0.5) | | Boklund et al. (2013) | |
| Probability to trace animal movements | | Constant = 0.98 | | Halasa et al. (2015) | |
| Probability to trace abattoir contact and survey the herd | | Constant = 0.88 | | Halasa et al. (2015) | |
| Probability to trace medium risk contact and survey the herd | | Constant = 0.4 | | Halasa et al. (2015) | |
| Probability to trace low risk contact and survey the herd | | Constant = 0.25 | | Halasa et al. (2015) | |
| Number of days required to trace animal shipments | | Uniform (min=0, max=2) | | Boklund et al. (2013) | |
| Number of days required to trace medium risk contacts | | Uniform (min=1, max=2) | | Boklund et al. (2013) | |
| Number of days required to trace low risk contacts | | Uniform (min=0, max=4) | | Boklund et al. (2013) | |
| Proportion of herds on the protection zone to be sampled for laboratory analysis during first visit | | Default = 0% | | The Danish Veterinary and Food Administration | |
| Proportion of herds on the surveillance zone to be sampled for laboratory analysis during first visit | | Default = 0% | | The Danish Veterinary and Food Administration | |
| Proportion of herds on the protection zone to be sampled for laboratory analysis during second visit | | 100% | | The Danish Veterinary and Food Administration | |
| Proportion of herds on the surveillance zone to be sampled for laboratory analysis during second visit | | Default = 0% | | The Danish Veterinary and Food Administration | |
| Duration of the protection zone | | 50 days | | The Danish Veterinary and Food Administration | |
| Duration of the surveillance zone | | 45 days | | The Danish Veterinary and Food Administration | |
| Scheduled first surveillance visit for herds in the protection zone | | 2 days | | The Danish Veterinary and Food Administration | |
| Scheduled second surveillance visit for herds in the protection zone | | 45 days | | The Danish Veterinary and Food Administration | |
| Scheduled surveillance visit for herds in the protection zone | | 40 days | | The Danish Veterinary and Food Administration | |
| Delay for a new surveillance visit for herds in overlapping zones | | 7 days | | The Danish Veterinary and Food Administration | |
| Transmission rate within nucleus and production herds | | PERT (min=0.14, mode=0.38,max=0.8) | | Re-estimated for between-pen transmission from Guinat et al. (2015) | |
| Transmission rate within boar stations, backyard, quarantine stations and hobby herds | | PERT (min=0.36, mode=0.60,max=0.93) | | Re-estimated for within-pen transmission from Guinat et al. (2015) | |
| Proportion of herds selected for laboratory testing from indirect tracing | | Default=10% | | The Danish Veterinary and Food Administration | |
| Infectiousness of subclinical cases compared to clinical cases | | Default=10% | | Assumption | |
| Probability of death following infection | | Default=95% | | Gallardo et al. (2015) | |
| Maximum number of days leftovers are infectious | | 5 | | Davis et al. (2015) | |
| Infectiousness of leftovers up to the maximum number of days leftovers can be infectious | | 100% | | Assumption | |
| Cumulative proportion of sick and died animals, over the normal level, before detection can be reached | | 0.0255 | | Estimated using mortality data from a large herd diagnosed with ASF in eastern Europe | |
| Cumulative number of sick or dead animals over the normal level, before first detection can be reached | | 5 | | Assumption | |
| Cumulative number of sick or dead animals over the normal level, before detection can be reached following first detection | | 1 | | Assumption | |

References:

Boklund A., L. Alban, N. Toft, Å. Uttenthal, 2009. Comparing the epidemiological and economic effects of control strategies against classical swine fever in Denmark. Prev Vet Med 90, 180-193.

Boklund, A., Halasa, T., Christiansen, L.E., Enøe, C., 2013, Comparing control strategies against foot-and-mouth disease: Will vaccination be cost-effective in Denmark? Prev. Vet. Med. 111, 206-219.

Gallardo, C., Soler, A., Nieto, R., Cano, C., Pelayo, V., Sanchaz, M.A., Pridotkas, G., Fernandez-Pinero, J., Briones, V., Arias, M., 2015. Experimental infection of domestic pigs with African swine fever virus Lithuania 2014 Genotype II field isolate. Transbound. Emerg. Dis. doi: 10.1111/tbed.12346.

Guinat, C., Gubbins, S., Vergne, T., Gonzales, J.L., Dixon, L., Pfeiffer, D.U., 2015. Experimental pig-to-pig transmission dyanmics for African swine fever virus, Georgia 2007/1 strain. Epidemiol. Inf. doi: 10.1017/S0950268815000862.

Halasa, T., Toft, N., Boklund, A., 2015. Improving the effect and efficiency of FMD control by enlarging protection or surveillance zones. Front. Vet. Sci. 2:70. doi: 10.3389/fvets.2015.00070.

Halasa, T., Bøtner, A., Mortensen, S., Christensen, H., Toft, N. and Boklund, A. (2016) Simulating the epidemiological and economic effects of an African swine fever epidemic in industrialized swine populations. Vet. Microbiol. 193, 7-16.

Nigsch, A., Costard, S., Jones, B.A., Pfeiffer, D.U., Wieland, B., 2013. Stochastic spatio-temporal modeling of African swine fever spread in the European Union during the high risk period. Prev. Vet. Med. 108, 262-275.
